#  Combined treatment with inhibitors of ErbB Receptors and Hh signaling pathways is more effective than single treatment in reducing the growth of malignant mesothelioma both in vitro and in vivo

**DOI:** 10.1186/s12967-022-03490-9

**Published:** 2022-06-25

**Authors:** Roberto Bei, Monica Benvenuto, Chiara Focaccetti, Sara Fazi, Marta Moretti, Daniela Nardozi, Valentina Angiolini, Sara Ciuffa, Loredana Cifaldi, Raffaele Carrano, Camilla Palumbo, Martino Tony Miele, Riccardo Bei, Giovanni Barillari, Vittorio Manzari, Enrico De Smaele, Andrea Modesti, Laura Masuelli

**Affiliations:** 1 Department of Clinical Sciences and Translational Medicine, University of Rome “Tor Vergata”, via Montpellier 1, 00133 Rome, Italy; 2 Saint Camillus International University of Health and Medical Sciences, via di Sant’Alessandro 8, 00131 Rome, Italy; 3 Department of Experimental Medicine, University of Rome “Sapienza”, viale Regina Elena 324, 00161 Rome, Italy; 4 Academic Department of Pediatrics (DPUO), Ospedale Pediatrico Bambino Gesù, IRCCS, 00165 Rome, Italy; 5 Department of Experimental Medicine, University of Rome “Tor Vergata”, Via Montpellier 1, 00133 Rome, Italy; 6 Medical School, University of Rome “Tor Vergata”, 00133 Rome, Italy

**Keywords:** Malignant mesothelioma, GANT-61, Afatinib, Hedgehog/GLI pathway, EGFR/ErbB receptors

## Abstract

Malignant mesothelioma (MM) is a rare orphan aggressive neoplasia with low survival rates. Among the other signaling pathways, ErbB receptors and Hh signaling are deregulated in MM. Thus, molecules involved in these signaling pathways could be used for targeted therapy approaches. The aim of this study was to evaluate the effects of inhibitors of Hh- (GANT-61) and ErbB receptors (Afatinib)-mediated signaling pathways, when used alone or in combination, on growth, cell cycle, cell death and autophagy, modulation of molecules involved in transduction pathways, in three human MM cell lines of different histotypes. The efficacy of the combined treatment was also evaluated in a murine epithelioid MM cell line both in vitro and in vivo. This study demonstrated that combined treatment with two inhibitors counteracting the activation of two different signaling pathways involved in neoplastic transformation and progression, such as those activated by ErbB and Hh signaling, is more effective than the single treatments in reducing MM growth in vitro and in vivo*.* This study may have clinical implications for the development of targeted therapy approaches for MM.

## Introduction

Malignant mesothelioma (MM) is a rare and aggressive tumor which mainly originates from pleural and peritoneal mesothelial cells [1, 2]. MM is histologically classified in three main subtypes: the epithelioid subtype, which has the best prognosis among the three, the sarcomatoid subtype, with the worst prognosis, and the biphasic or mixed subtype, with both epithelioid and sarcomatoid features [3]. Untreated pleural MM patients have a median survival time of 6 months, and the majority of patients die within 24 months after diagnosis [1, 2]. The median overall survival of pleural MM with a single chemotherapeutic agent is 7–8 months and only few drugs have a response rate of 15–20% [2]. The current standard therapy for MM is the combination of pemetrexed and cisplatin chemotherapy with a response rate of ~ 40% [1, 4]. Anti-angiogenic therapy was shown to enhance overall survival when added to the first line therapy [1, 5]. It has been recently shown that the application of hyperthermic intraperitoneal chemotherapy (HIPEC) and cytoreductive surgery increased MM patients’ survival in particular for peritoneal MM [6, 7]. However, the therapeutic strategies for the treatment of pleural MM are referred to as ‘life-extending treatments’ [8]. Recent findings on the pathogenesis of MM have emphasized the importance of tumor suppressor gene alterations for sustaining aberrant signaling pathways which promote the uncontrolled growth of mesothelial cells [9–12]. In agreement with new findings, with awareness of the resistance of MM to conventional therapies and of the poor patient survival following traditional chemotherapy, novel molecular targeted therapies for MM treatment have been identified [2, 12–14]. Novel therapeutic approaches include inhibitors of mTOR, folate, receptor tyrosine kinases, ciclooxygenase and angiogenesis, synthetic lethal treatment, miRNA replacement, oncoviral therapies, and immunotherapy alone or in combination with chemotherapy [2, 8, 14, 15]. Among the other aberrantly activated signaling pathways in MM, it has been reported that the EGF receptor (EGFR) is overexpressed in approximately 60% of human pleural MMs, but is not expressed in normal pleura [16, 17]. In addition, at least one ErbB family member was found to be expressed in 88% of tumors. ErbB receptor expression was strongly dependent upon histologic subtype, with highest expression in epithelioid tumors [17, 18]. It was found that MMs expressed EGFR (79.2%), ErbB4 (49.0%) and HER2 (6.3%), but lacked ErbB3 [18]. Recently, a patient with pleural MM harboring both G719C and S768I EGFR mutations was successfully treated with Afatinib (AFA), a second-generation EGFR/HER tyrosine kinase inhibitor (TKI) [19]. However, other clinical studies using EGFR TKIs in MM have not reported clinical efficacy [17, 20, 21]. Mechanisms of resistance to EGFR inhibition by TKIs could be due to a simultaneous activation of alternative signaling pathways and rare mutations of EGFR in MM [22, 23]. On the other hand, it has been suggested that when chemotherapy options have been exhausted, the use of EGFR TKIs is indicated in patients with wild-type EGFR tumors [24]. Finally, it was reported that AFA induced apoptosis in non-small cell lung carcinoma (NSCLC) cells without EGFR mutation [25].

The Hedgehog (Hh)/Glioma-associated oncogene (GLI) pathway is a complex signaling pathway which carries out critical functions in vertebrate embryogenesis and adult tissue homeostasis [26, 27]. There are three Hh homologs in vertebrates: Sonic Hedgehog (Shh), Indian Hedgehog (Ihh) and Desert Hedgehog (Dhh) [28]. GLI family of zinc-finger transcription factors and Smoothened (SMO) are signal transducers of the Hh pathway frequently aberrantly activated in tumors and MMs [29–31]. After ligand binding, SMO enters the cilium and transduces the Hh signal, by activating the cytoplasmic GLI transcription factors. GLI1 and GLI2 stimulate the function of Shh-GLI1/2 while GLI3 antagonizes them [28, 32]. High GLI1 and SMO expression levels and aberrant activation were associated with poor survival in patients with pleural MM [30, 33]. SMO and GLI-related inhibitors have shown anti-cancer properties both in vitro and in vivo and when employed in clinical trials [31]. However, the resistance to Hh signaling pathway inhibitors remains a drawback to overcome [31].

Given these premises, the aim of this study was to evaluate the combined effect of inhibitors of the Hh- (GANT-61) and ErbB receptors (AFA)-mediated signaling pathways on the growth of MM both in vitro and in vivo. We demonstrate that combined treatment with both inhibitors is more effective than the single treatments in reducing the growth of MM. Therefore, the combined use of two drugs capable of counteracting the activation of two different aberrantly activated signaling pathways could be a useful tool to reduce the growth of MM cells.

## Materials and methods

### Reagents

DMSO and Sulforhodamine B (SRB) were purchased from Sigma–Aldrich (Milan, Italy). Afatinib (AFA) and GANT-61 (GANT) were obtained from MedChem Express (Monmouth Junction, NJ, USA). Z-VAD-FMK was obtained from Calbiochem (San Diego, CA, USA). Antibodies against γ-H2AX (cat. no. 560443; 1:2000), ERK (cat. no. 51-9002015; 1:200), phospho-ERK (cat. no. 51-9001962; 1:200), JNK/SAPK1 (cat no. 610627; 1:250), JNK/SAPK (pT183/pY185) (cat. no. 612540; 1:250), p38a/SAPK2a (cat. no. 51-9002050; 1:1000), and p38 MAPK (pT180/pY182) (cat. no. 51-9002043; 1:1000) were obtained from BD Pharmingen (BD Biosciences, San Jose, CA, USA). Antibodies against caspase 8 (cat. no. #9746; 1:500) and activated caspase 3 (cat. no. #9661; 1:500) were obtained from Cell Signaling Technology (Danvers, MA, USA). Antibodies against Bax (cat. no. sc-493; 1.200), Bcl-2 (cat. no. sc-7382; 1:200), PARP-1 (F-2) (cat. no. sc-8007; 1:200), and AKT (cat. no. sc-8312; 1:200) were obtained from Santa Cruz Biotechnology (Santa Cruz, CA, USA). Anti-ErbB2 (1:1000) and anti-EGFR (1:1000) antisera were provided by Dr. M. H. Kraus (University of Alabama, Birmingham, AL, USA) [34]. Antibodies against Beclin-1 (cat. no. ab62557; 1:1000) and SQSTM-1/p62 (cat. no. ab109012; 1:1000) were obtained from Abcam (Cambridge, United Kingdom) and the anti-LC3 antibody (cat. no. NB-100-2220; 1:500) was purchased from Novus Biologicals (Littleton, CO, USA). Antibody against tubulin (cat. no. MAB-94264; 1:5000) was purchased from Immunological Sciences (Rome, Italy). Rabbit polyclonal anti-actin (cat. no. A5060; 1:500), the goat anti-mouse (cat. no. A4416; 1:5000) or -rabbit (cat. no. A6154; 1:10,000) IgG peroxidase conjugated secondary antibodies were obtained from Sigma-Aldrich.

### Cell lines and treatments

Human MM cell lines (H-Meso-1, MM-F1, MM-B1) were kindly provided by Prof. Antonio Procopio (Università Politecnica delle Marche, Ancona, Italy) and previously described [35, 36]. The murine MM cell line #40a was kindly provided by Dr. Agnes Kane (Department of Pathology and Laboratory Medicine, Brown University, Providence, RI, USA). The cell lines were grown at 37 °C in a humidified incubator with an atmosphere of 5% CO_2_. Isolation of the murine MM 40-cell line was previously described by Goodglick et al. [37]. The #40a cell line is derived from the 40-cell line after two passages in the peritoneal cavity of syngeneic C57BL/6 mice following administration of pristane one week before cells transplant. These passages allow the selection of cells which reproducibly form ascites when intraperitoneally injected in mice. H-Meso-1 cells have an epithelial morphology, while MM-F1 and MM-B1 cells have sarcomatous and biphasic features, respectively [38]. The 40-cell line has an epithelial morphology [37]. AFA and GANT were dissolved in DMSO. For treatments, cells were incubated for the indicated times in the presence of vehicle control (DMSO ≤ 0.1%) and AFA (2.5-5-10 μM) or GANT (2.5-5-10-20 μM), alone or in combination.

### Sulforhodamine B (SRB) assay

SRB assay was used to investigate cell survival, by measuring the cellular protein content of adherent cultures. SRB is a dye which stoichiometrically binds to basic amino acids under mild acidic conditions and dissociates using basic conditions [39]. Cells were seeded at 5 × 10^3^/well in 96-well plates and incubated at 37 °C to allow cell attachment. After 24 h, the medium was changed and the cells were treated with AFA (2.5-5-10 µM), GANT (2.5-5-10-20 µM) or DMSO, and AFA + GANT (2.5 µM + 2.5 µM; 5 µM + 5 µM; 10 µM + 10 µM; 10 µM + 20 µM) and incubated for 24, 48 and 72 h. The cells were then fixed with 50 µl/well of 50% cold trichloroacetic acid (TCA) for 1 h at 4 °C. After four washes with distilled water, plates were air-dried and stained for 30 min with 100 µl/well of 0.4% (wt/vol) SRB in 1% acetic acid. After four washes with 1% acetic acid to remove the unbound dye, plates were air-dried, and cell-bound SRB was dissolved with 100 µl/well of 10 mM pH 10 unbuffered Tris base solution [40]. The optical density (O.D.) of the samples was determined at 540 nm with a spectrophotometric plate reader. The percentage of cell survival was determined considering the O.D. values of the samples treated with AFA and GANT, alone and in combination, compared to those of the samples treated with DMSO [41, 42]. Experiments were performed in triplicate and repeated two times.

The combined effects of GANT and AFA were analysed according to the method of Kern, as previously reported. According to this method, cell survival data were processed to obtain an index (R) defined as follows: R = S_exp_/S_obs_, where S_exp_, the expected survival, is the product of the percentage survival observed with AFA alone and the percentage survival observed with GANT alone, and S_obs_, the observed survival, is the actual percentage survival observed with the AFA + GANT combination.

An R index lower than 1 indicates that the combination exerts a less than additive effect; an R index of 1 indicates that the effect is additive, and any value of R greater than unity indicates a synergistic interaction [43, 44].

### Trypan Blue exclusion test

For trypan blue exclusion test, cells were seeded at 5 × 10^4^/well in 24-well plates and incubated at 37 °C to allow cells attachment. After 24 h, the medium was changed and the cells were treated with AFA (2.5-5-10 µM), GANT (2.5-5-10-20 µM) or DMSO, and AFA + GANT (2.5 µM + 2.5 µM; 5 µM + 5 µM; 10 µM + 10 µM; 10 µM + 20 µM). After 24, 48, and 72 h, adherent as well as suspended cells of each well were harvested and stained with trypan blue (Sigma-Aldrich, Milan, Italy) and counted with an optic microscope [45, 46]. Experiments were performed in triplicate and repeated two times. Percentage of cells death was determined compared to the total number of cells [47, 48].

### FACS analysis

Asynchronized, log-phase growing cells (60% confluent, approximately 2.5 × 10^5^/well in 6-well plates) were treated with AFA (5 and 10 µM), GANT (10 and 20 µM) and AFA + GANT (10 µM + 20 µM) or DMSO in complete culture medium. Z-VAD-FMK was used at a final concentration of 40 μM for 2 h before adding the treatments. After 48 h, adherent as well as suspended cells were harvested, centrifuged at 1500 rpm for 10 min and washed twice with cold phosphate-buffered saline (PBS). Cell pellets were re-suspended in 70% ethanol and incubated for 1 h at −20 °C. Cells were then washed twice with cold PBS, centrifuged at 1500 rpm for 10 min, incubated for 1 h in the dark with propidium iodide (25 µg/ml final concentration in 0.1% citrate and 0.1% Triton X-100) and analyzed by flow cytometry using a FACSCalibur cytometer with CellQuest software [47, 49].

### Western blotting

About 1.5 × 10^6^ cells were seeded in 100 mm tissue culture dishes 24 h prior to the addition of AFA 10 μM, GANT 20 μM, AFA 10 μM + GANT 20 μM or DMSO. After 48 h of treatment, cells were harvested, washed twice with cold PBS, and lysed in RIPA buffer as previously described [44]. For immunoblotting analysis, 80 μg of cell lysates were resolved in 10–12% SDS-PAGE and then transferred to nitrocellulose membranes [50]. After blocking, the membranes were incubated with specific primary antibodies at 1–2 μg/ml concentrations overnight at 4 °C. Equal loading and transfer of proteins was verified by Ponceau red staining of the membranes and by analyzing actin or tubulin expression. The assay was then performed as previously described, and signals were detected using enhanced chemiluminescence system ECL LiteAblot (Euroclone, Milan, Italy) [51, 52].

The densitometric analysis of bands was performed with the ImageJ 1.53e software (NIH, Bethesda, MD, USA) after blot scanning and expressed as bar graphs in the figures.

### RNA extraction and RT-qPCR

About 1.5 × 10^6^ cells were seeded in 100 mm tissue culture dishes 24 h prior to the addition of AFA 10 μM, GANT 20 μM, AFA 10 + GANT 20 μM or DMSO. After 24 h, total mRNA was extracted using TRizol (Invitrogen-Thermo Fisher Scientific, Waltham, CA, USA) and RNA Clean & ConcentratorTM-5 (R1014, Zymo Research, Irvine, CA, USA) according to manufacturer’s instructions. cDNA synthesis was performed using the High-Capacity cDNA reverse transcription kit (BIO-65054, Meridian Bioscience, Cincinnati, OH, USA). Quantitative real-time PCR analysis of specific mRNA levels (GLI1, GLI2, Ptch1) was performed on cDNAs employing TaqMan gene expression assay (Applied Biosystem, Thermo Fisher Scientific) and using the ViiATM7 Real-Time PCR System (Applied Biosystem, Thermo Fisher Scientific). Primers for gene expression were Hs00171790_m1 GLI1, Hs01119974_m1 GLI2, Hs00181117_m1 Ptch1 (Applied Biosystem, Thermo Fisher Scientific). Experiments were biologically replicated at least three times, and all of them were performed with three technical replicates. Relative mRNA expression was normalized on the mean of expression of four housekeeping genes (GAPDH, TBP, HPRT and β-Actin) and it was calculated using the ΔΔCt method as in Spiombi et al*.* [53].

### Treatment of C57BL/6 mice with AFA and GANT, alone and in combination

Groups of 6-to-8-weeks-old female C57BL/6 mice (8 mice for each group) were intraperitoneally (i.p.) inoculated with 0.2 ml of suspension containing 1 × 10^6^ #40a cells in PBS. Mice were then divided into 4 experimental groups:

Group I: mice treated with AFA administered in the peritoneum (25 mg/kg body weight, dissolved in PBS + DMSO).

Group II: mice treated with GANT administered in the peritoneum (50 mg/kg body weight, dissolved in PBS + DMSO).

Group III: mice treated with AFA + GANT administered in the peritoneum (25 mg/kg + 50 mg/kg body weight, dissolved in PBS + DMSO).

Group IV: control mice treated with PBS + DMSO administered in the peritoneum.

Treatments were started simultaneously with the inoculation of MM cells. Isolation of the murine MM #40a cell line was previously described [37].

Investigation has been conducted in accordance with the ethical standards and according to the Declaration of Helsinki and the ARRIVE guidelines. A veterinary surgeon was present during the experiments. The animal care, both before and after the experiments, was performed only by trained personnel. Mice were bred under pathogen-free conditions in the animal facilities of the University of Rome “Tor Vergata” and handled in compliance with European Union (EU Directive 2010/63/EU) and institutional standards for animal research. The work was conducted with the formal approval of the local animal care committees (institutional and national), and animal experiments have been registered as legislation requires (Authorization from Ministry of Health no. 179_2020-PR).

### Analysis of antitumor activity in vivo

Growth of #40a cells in the C57BL/6 mice induces ascites. Accordingly, the abdominal circumference of mice was monitored before the inoculation of cells and every week until tumor-bearing mice were euthanized at the first signs of distress or when their abdominal circumference exceeded 12 cm [47].

### Statistical analysis

Data distribution of cell survival, cell death and FACS analysis was preliminarily verified using the Kolmogorov-Smirnov test, and the data sets were analyzed by one-way analysis of variance (ANOVA) followed by the Newman-Keuls test. Differences in the intensity of immunoreactive bands were evaluated by one-way ANOVA followed by the Newman-Keuls test. Values with p ≤ 0.05 were considered significant. Survival curves and tumor volumes were analyzed using the KaplanMeier method and compared with a log-rank test (Mantel-Cox). Differences in tumor volumes were regarded as significant when the p-value was p ≤ 0.05.

## Results

### Effect of GANT and AFA, alone or in combination, on human and mouse MM cell survival and death

**Fig. 1:**
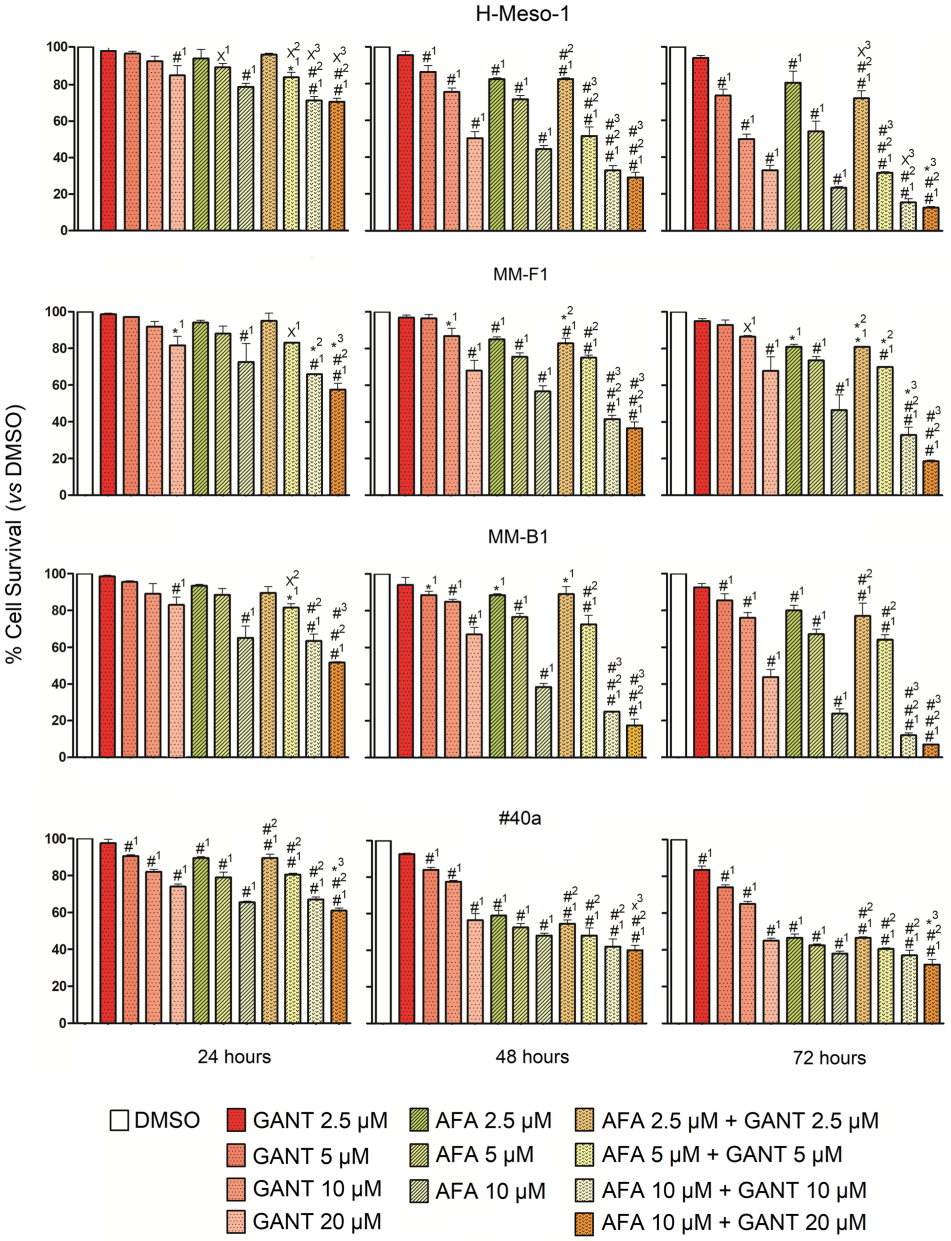
Effect of GANT-61 (GANT) and Afatinib (AFA), alone or in combination, on MM cell lines survival. The survival of human (H-Meso-1, MM-F1, MM-B1) and murine (#40a) cell lines was assessed by the SRB assay after 24, 48, and 72 h of treatment with DMSO or GANT or AFA, alone or in combination. The percentage of surviving cells treated with the compounds was calculated by normalizing the O.D. value to that of the control cultures (DMSO). The results are expressed as the mean ± SD of two independent experiments performed in triplicate. Statistical significance of the effects obtained with GANT and AFA, alone or in combination, was calculated *vs* those obtained in (1) DMSO-, (2) GANT-, and (3) AFA-treated cells with one-way ANOVA. ^×^p < 0.05; *p < 0.01; ^#^p < 0.001

The survival and death of human (H-Meso-1, MM-F1, MM-B1) and mouse (#40a) MM cells were evaluated by the SRB and Trypan blue exclusion assays, respectively, after exposure to increasing doses of GANT (2.5, 5, 10, 20 µM) and AFA (2.5, 5, 10 µM), alone or in combination (AFA + GANT, at each individual concentration), or vehicle control (DMSO) for 24, 48 and 72 h. The effects of GANT and AFA on cells survival and death were dose-dependent. However, while the effect of AFA in the SRB assay achieved statistical significance at all doses tested and in all cell lines compared to vehicle control treatment after 48 and 72 h, the effect of GANT at the lower doses was dependent on the cell lines (Fig. 1). The survival of human cell lines upon GANT treatment was not affected at the doses of 2.5 µM for MM-B1 and H-Meso-1, and 2.5 and 5 µM for MM-F1 after 48 and 72 h of treatment. The effect obtained with equimolar combinations of AFA + GANT was significantly higher than the effect of treatment with GANT at all concentrations on all cell lines after 72 h of treatment, while it was higher than AFA alone only at 10 µM on MM-B1 and MM-F1, and at 10 and 5 µM on H-Meso-1, after 48 and 72 h of treatment. Conversely, the effect obtained with GANT at 20 µM and AFA at 10 µM was significantly higher than the effect of treatment with GANT or AFA alone in all cell lines after 24, 48 and 72 h.

**Fig. 2:**
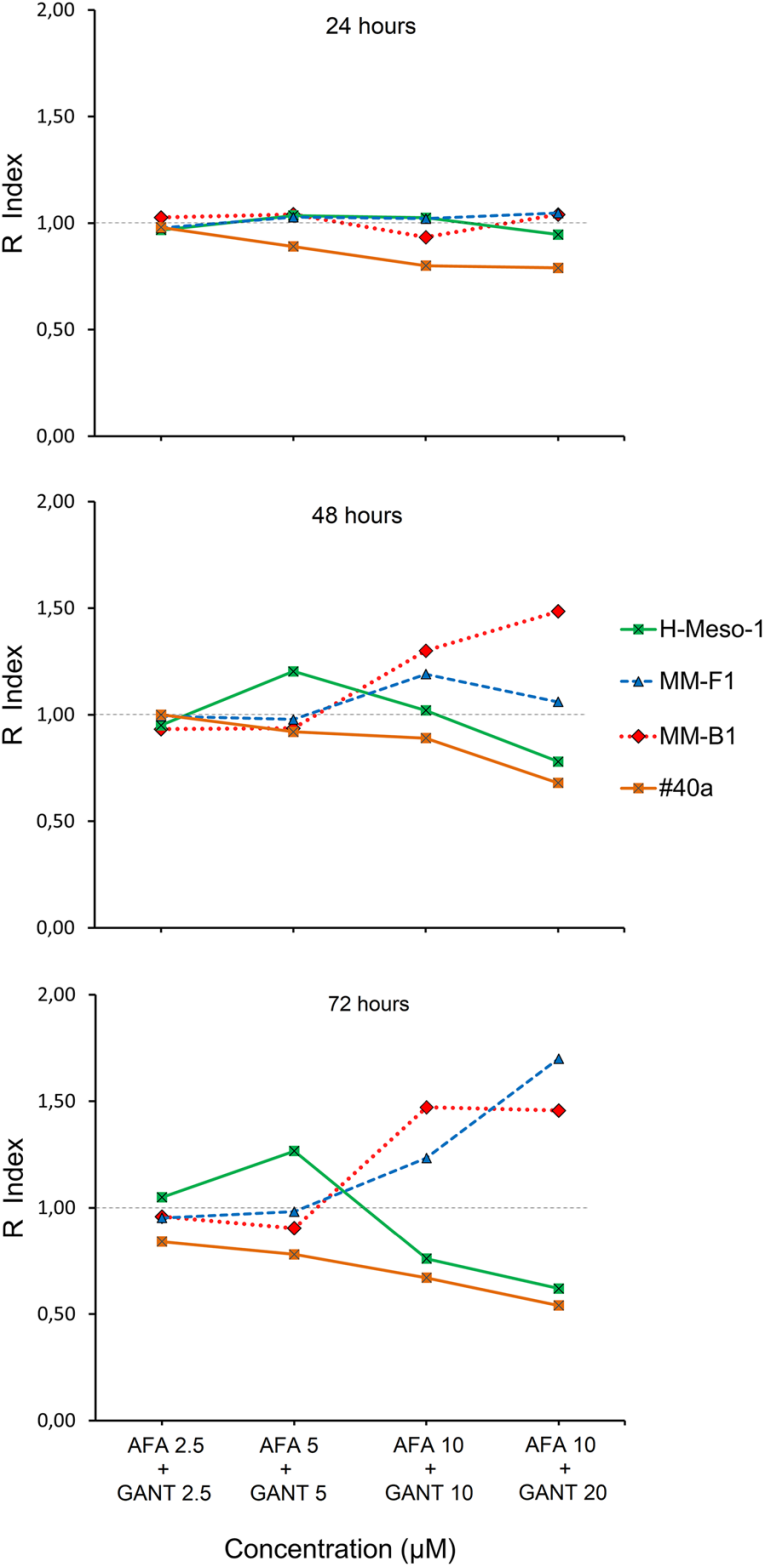
Interaction between GANT and AFA, as assessed by the analysis of cell survival of MM cell lines, according to the method of Kern. The figure represents Kern Index (R) values for the two drugs combined at the indicated concentrations. R > 1 represents a synergistic effect, R = 1 indicates that the effect is additive, R < 1 indicates that the effect of the combined treatment is less than additive

The mode of interaction between AFA and GANT when used in combination was determined using the method of Kern (Fig. 2). A Kern Index R > 1 represents a synergistic effect, R < 1 indicates that the effect of the combined treatment is less than additive, and R = 1 indicates that the effect is additive. In two cell lines, i.e. MM-F1 and MM-B1, the combination of AFA and GANT showed a synergistic effect when the two drugs were used at high concentrations (10 + 10 or 10 + 20 µM) for 48 and 72 h. Conversely, in H-Meso-1 cells treated for 48–72 h the combination of AFA and GANT showed a less than additive effect at high concentrations, while a synergistic effect was obtained at a dose of 5 + 5 µM. In the murine cell line #40a, a less than additive effect was observed with all combinations of AFA and GANT, except at 2.5 + 2.5 µM for 24 and 48 h.

**Fig. 3:**
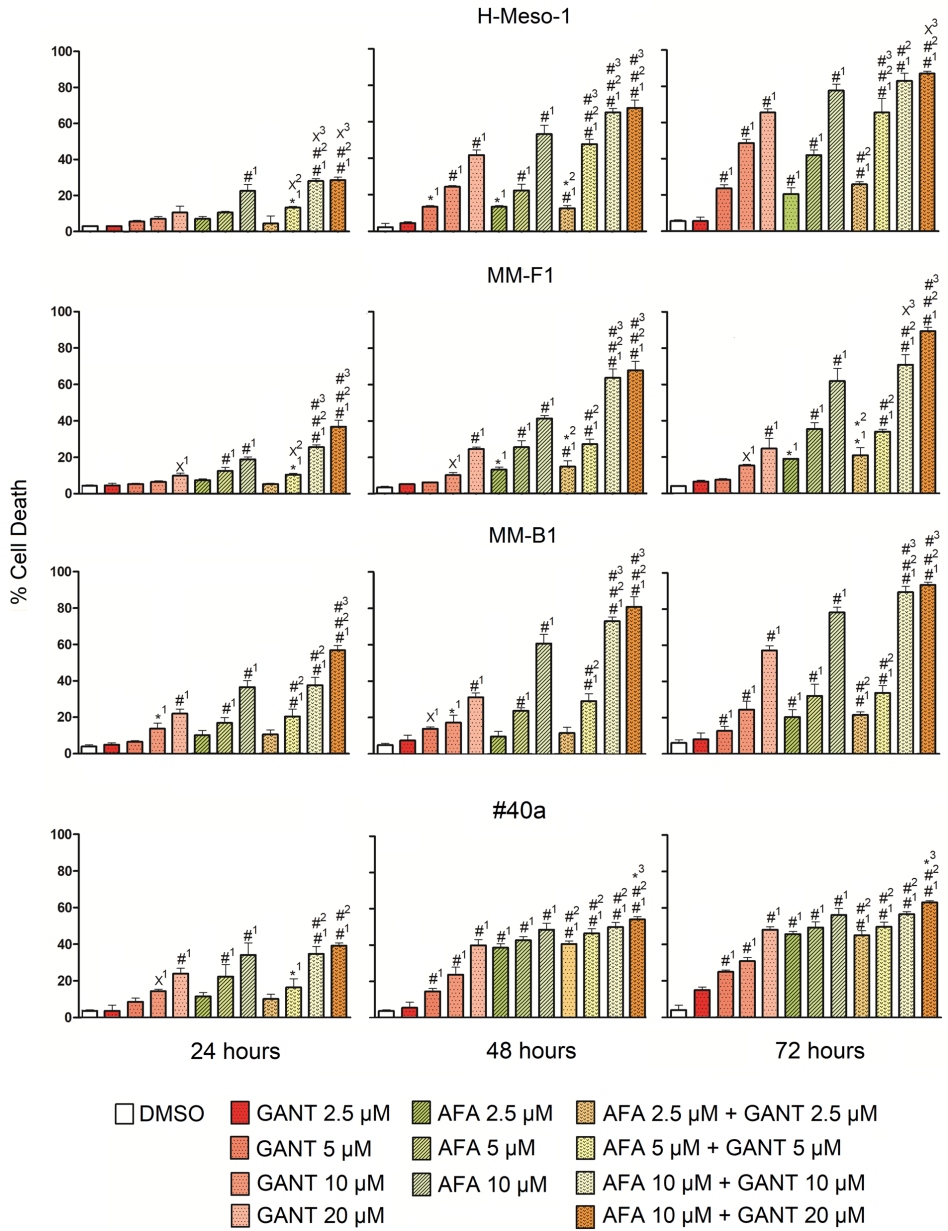
Effect of GANT and AFA, alone or in combination, on the death of MM cell lines. The death of human (H-Meso-1, MM-F1, MM-B1) and murine (#40a) cell lines was assessed by the Trypan blue assay after 24, 48, and 72 h of treatment with DMSO or GANT or AFA, alone or in combination. The results are expressed as the mean ± SD of two independent experiments performed in triplicate. Statistical significance of the effects obtained with GANT and AFA, alone or in combination, was calculated *vs* those obtained in (1) DMSO-, (2) GANT- and (3) AFA-treated cells with one-way ANOVA . ^×^p < 0.05; *p < 0.01; ^#^p < 0.001

Treatment with AFA 10 + GANT 20 μM significantly increased cells death rate compared to single treatments after 24, 48 and 72 hours in all human MM cell lines (Fig. 3). Conversely, the treatment with AFA 10 + GANT 20 μM was able to increase the rate of cell death compared to single treatments in the murine cell line #40a only after 48 and 72 hours.

### Effects of GANT and AFA, alone or in combination, on apoptosis and cell cycle distribution of human and mouse MM cell lines

**Fig. 4:**
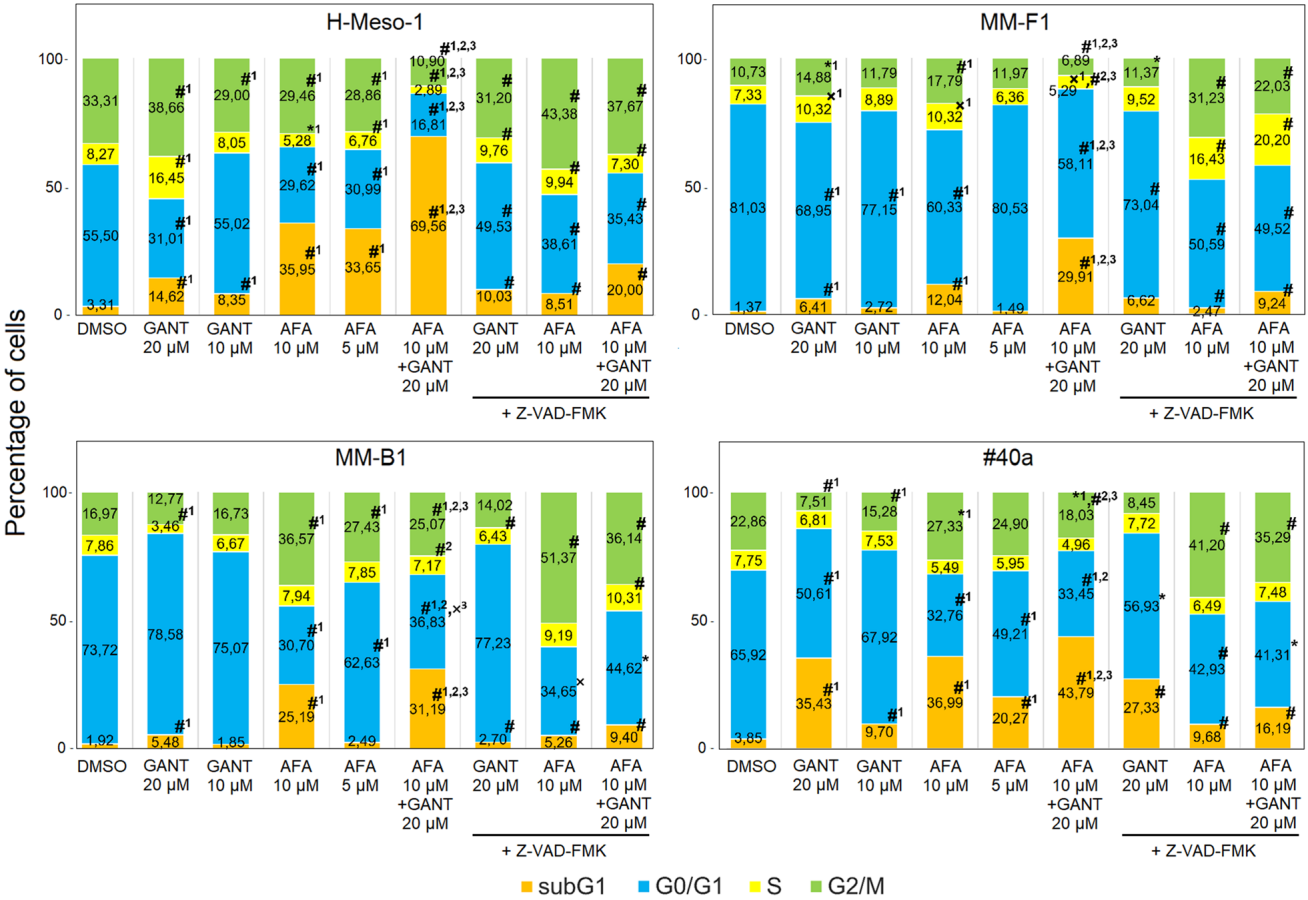
Effects of GANT and AFA, alone or in combination, on cell cycle distribution. Stacked bar graphs show the percentage of cells in subG1, G0/G1, S, and G2/M phases, as calculated with CellQuest Pro 5.2 software. Results represent mean values from two independent experiments. Statistical significance of the effects obtained with GANT and AFA, alone or in combination, was calculated *vs* those obtained in (1) DMSO-, (2) GANT- and (3) AFA-treated cells with one-way ANOVA. The statistical significance of the effect obtained with GANT 20, AFA 10, or AFA 10 + GANT 20 in the presence of the Z-VAD-FMK inhibitor (Z-VAD) was calculated with respect to that obtained in cells treated with GANT 20, AFA 10, or AFA 10 + GANT 20, respectively. ^×^p < 0.05; *p < 0.01; ^#^p < 0.001

To evaluate the effect of GANT and AFA, alone or in combination, on the induction of apoptosis and on the modulation of the cell cycle, FACS analysis of the DNA content was performed. H-Meso-1, MM-F1, MM-B1 and #40a cells were treated for 48 h with increasing doses of GANT (10, 20 μM) and AFA (5, 10 μM), alone or in combination. As shown in Fig. 4, GANT (20 μM) and AFA (10 μM), when used alone, were able to significantly increase the percentage of cells in the subG1 phase, compared to DMSO, in all cell lines. The combined AFA 10 + GANT 20 µM treatment was more effective in increasing the number of cells in the subG1 phase than the single treatments in all cell lines. This increase was paralleled by a variable decrease in the number of cells in the other cell cycle phases in H-Meso-1, MM-F1 and #40a cell lines and by the decrease and increase in the percentage of cells in G0/G1 and G2/M phases, respectively, in MM-B1 cells. To confirm the induction of apoptosis, cells were simultaneously treated with GANT, AFA or AFA + GANT (10 + 20 μM), and with the universal caspase inhibitor, Z-VAD-FMK. Z-VAD-FMK significantly reduced the number of subG1 phase-cells in all treatment conditions and cell lines, except in the GANT-treated MM-F1 cell line. On the whole, this result corroborated the induction of apoptosis following single and combined treatments in these MM cells (Fig. 4).

### Effects of GANT and AFA, alone or in combination, on the expression of molecules involved in apoptosis in MM cell lines

**Fig. 5:**
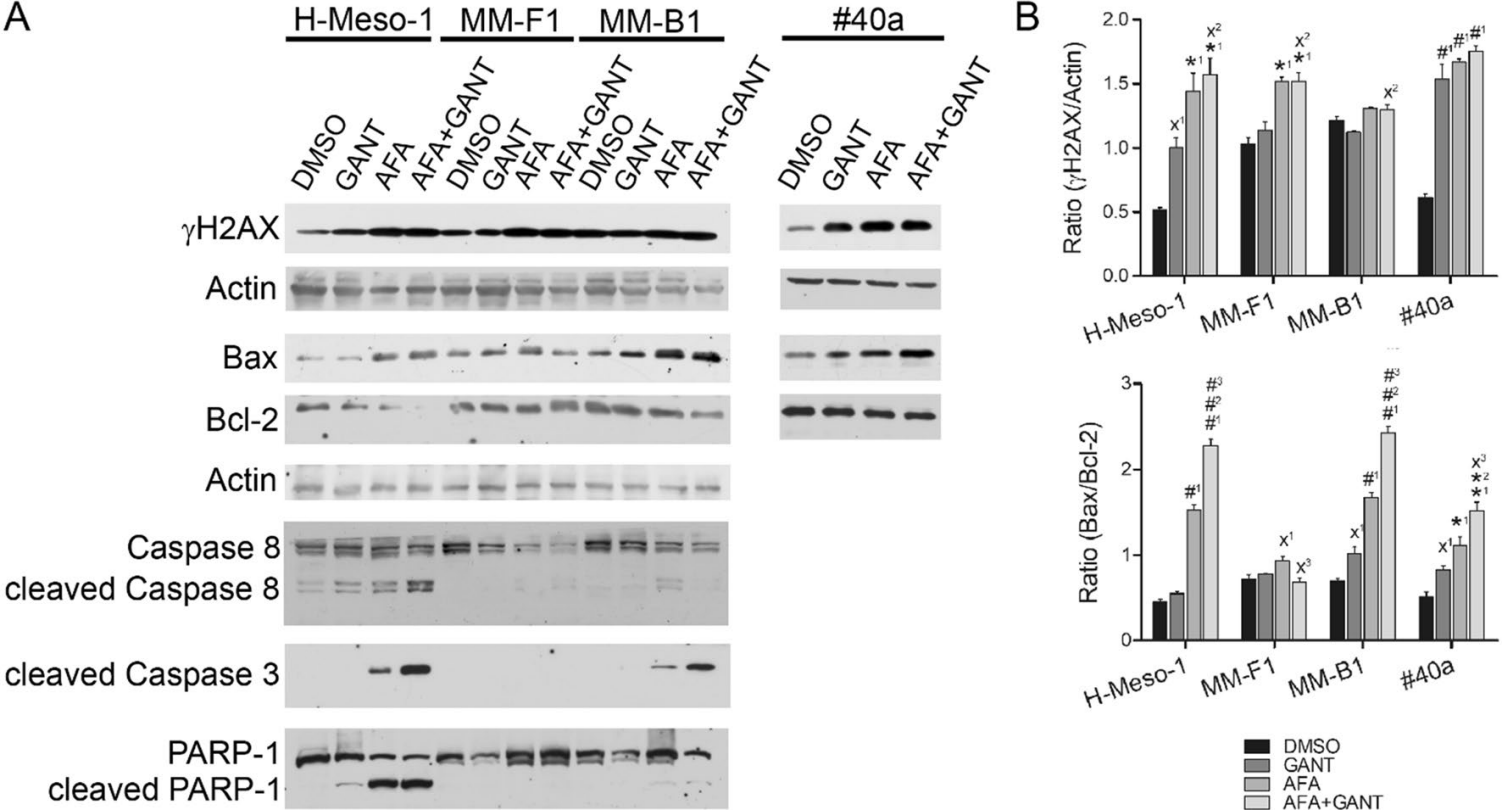
Effects of GANT and AFA, alone or in combination, on molecules involved in apoptosis in MM cell lines. **A** The expression of Bax, Bcl-2, caspase 8, caspase 3, PARP-1 and γH2AX was evaluated by Western blotting analysis following treatment for 48 h with AFA 10 μM, GANT 20 μM, AFA 10 μM + GANT 20 μM, or DMSO. Actin was used as internal control. **B** The densitometric ratios between γH2AX and actin, Bax and Bcl-2, and statistical analysis are reported. The intensity of the bands was quantified using the ImageJ 1.53e software after blot scanning. Statistical significance of the effects obtained with GANT and AFA, alone or in combination, was calculated *vs* those obtained in (1) DMSO-, (2) GANT- and (3) AFA-treated cells with one-way ANOVA (˟p ≤ 0.05, *p ≤ 0.01, ^#^p ≤ 0.001). Data are expressed as the mean ± SD of two independent experiments. Cropped images were used for Western Blotting

To corroborate the induction of apoptosis following single or combined treatments with AFA and GANT, the expression of Bax, Bcl-2, procaspase, and caspase 8/3, and cleavage of the poly (ADP-ribose) polymerase-1 (PARP-1) was analyzed by Western blotting in MM-treated cells (Fig. 5). The Bax/Bcl-2 ratio was increased by GANT in MM-B1 and #40a cells, and by AFA in all cell lines. Of note, the combined AFA + GANT treatment was more efficient than AFA alone in increasing Bax/Bcl-2 ratio in three cell lines, i.e. H-Meso-1, MM-B1 and #40a (Fig. 5).

An increase in Bax/Bcl-2 ratio promotes apoptosis, by determining the activation of initiator caspases and finally of caspase 3. The expression and activation of the initiator caspases 8 and the expression of activated caspase 3 was thus determined in MM cells upon treatments. Procaspase 8 cleavage in the activated fragments p43/41 was observed in AFA- and AFA + GANT-treated H-Meso-1, MM-F1 and MM-B1 cell lines (Fig. 5A). Moreover, a decrease of procaspase 8 expression was evident upon AFA and AFA + GANT treatments in MM-F1 and MM-B1 cells (Fig. 5A). Furthermore, activation of caspase 3 was observed in H-Meso-1 and MM-B1 cell lines treated with AFA, and the addition of GANT potentiated the activation of caspase 3 in these cell lines (Fig. 5A). The activated caspase 3 induces the proteolytic cleavage of the PARP-1 enzyme thus inhibiting DNA repair. AFA and AFA + GANT induced PARP-1 cleavage in H-Meso-1 and MM-B1 cell lines (Fig. 5A), as shown by the appearance of the 89 kDa proteolytic fragment. GANT alone was able to induce PARP-1 cleavage only in the H-Meso-1 cell line. The different treatments did not induce caspase 3 activation or PARP-1 cleavage in MM-F1 cells (Fig. 5A).

The phosphorylation of histone H2AX at Ser139 occurs in response to DNA double-strand break (DSB) formation and is required during the activation of the apoptotic process for the condensation of chromatin. The phosphorylated form of H2AX is called γH2AX [54, 55]. The analysis of γH2AX in AFA-, GANT- and AFA + GANT-treated cells revealed a significant increase in γH2AX in H-Meso-1 and #40a cell lines. AFA and AFA + GANT increased γH2AX in MM-F1 cells thus supporting activation of apoptosis in this cell line (Fig. 5).

### Effects of GANT and AFA, alone or in combination, on autophagy in MM cells

Autophagy is one of cells modality to respond to stressors. To demonstrate whether AFA 10 μM, GANT 20 μM and AFA 10 μM + GANT 20 μM treatments were able to induce autophagy in MM cell lines, the expression of proteins involved in the autophagic pathway, including Beclin-1, SQSTM-1/p62 and LC3-I/II was analyzed by Western blotting.

**Fig. 6:**
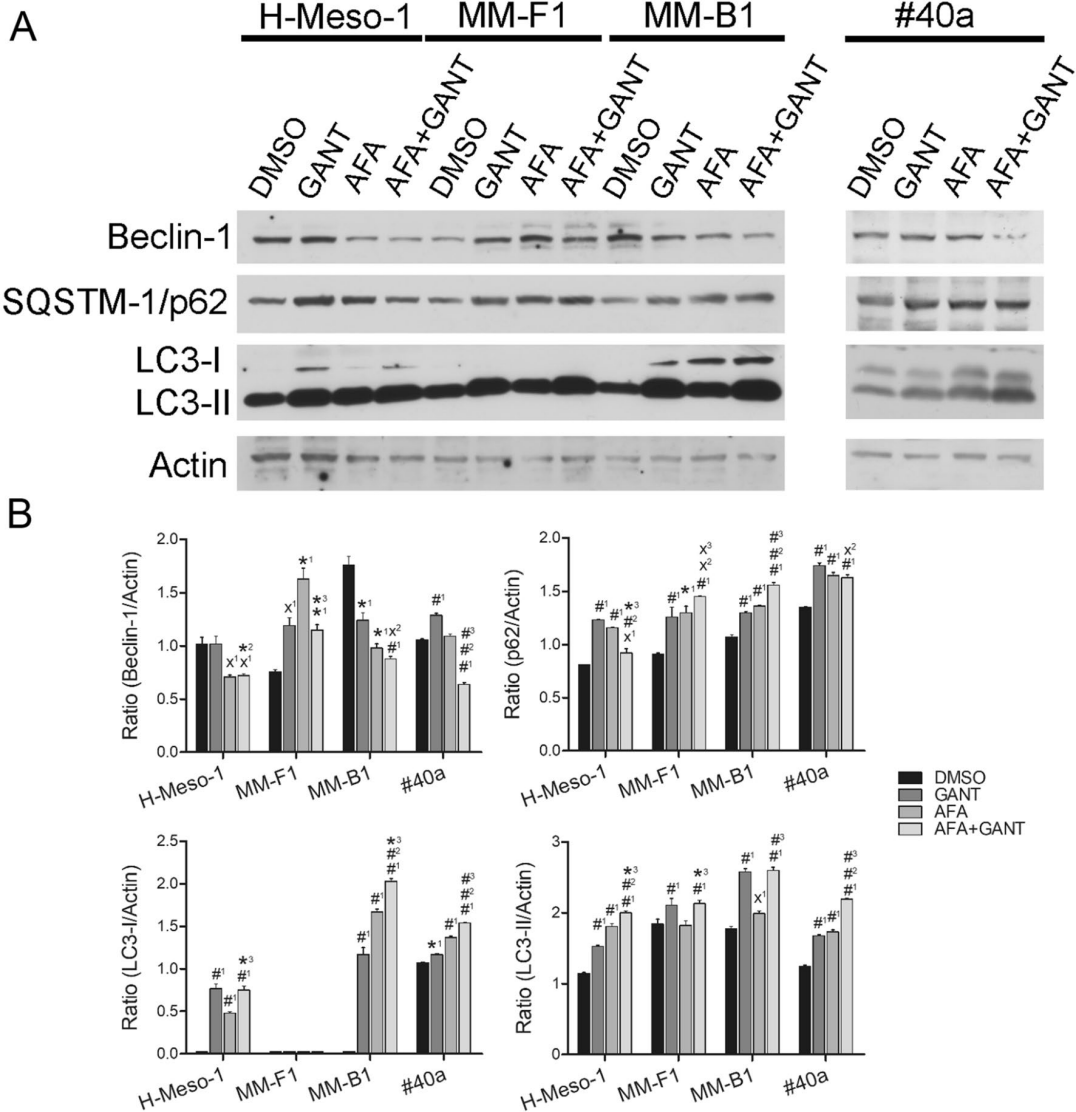
Effects of GANT and AFA, alone or in combination, on autophagy in MM cell lines. **A** Western blotting analysis was performed on MM cell lines treated for 48 h with AFA 10 μM, GANT 20 μM, AFA 10 μM + GANT 20 μM, or DMSO. Actin was used as an internal control. **B** Densitometric ratios and statistical analysis are reported. The intensity of the bands was quantified using the ImageJ 1.53e software after blot scanning. Statistical significance of the effects obtained with GANT and AFA, alone or in combination, was calculated *vs* those obtained in (1) DMSO-, (2) GANT- and (3) AFA-treated cells with one-way ANOVA (˟p ≤ 0.05, *p ≤ 0.01, ^#^p ≤ 0.001). Data are expressed as the mean ± SD of two independent experiments. Cropped images were used for Western Blotting

Beclin-1 levels were decreased in H-Meso-1 and MM-B1 cell lines after treatment with AFA when used alone and in combination with GANT, as compared to DMSO-treated cells; in addition, GANT potentiated the decrease of Beclin-1 induced by AFA in MM-B1 cells, although not significantly compared to AFA single treatment. On the other hand, AFA + GANT treatment induced a significant decrease of Beclin-1 in #40a cells, as compared to DMSO-, GANT- and AFA-treated cells (Fig. 6). An increase in Beclin-1 was instead observed in MM-F1 cells treated with AFA and GANT, alone or in combination, as compared to DMSO-treated cells.

An increased conversion of LC3-I to LC3-II indicates the formation of autophagosomes. LC3-II was constitutively expressed in DMSO-treated MM cell lines, while LC3-I was constitutively expressed only in #40a cells. All treatments increased the expression of LC3-II, as compared to DMSO-treated cells and induced the expression of LC3-I in H-Meso-1, MM-B1 and #40a cells. Only GANT and AFA + GANT increased the expression of LC3-II in MM-F1 cells (Fig. 6). In addition, the levels of SQSTM-1/p62 increased in GANT- and AFA-treated H-Meso-1, MM-F1, MM-B1 and #40a cells. AFA + GANT also increased the accumulation of SQSTM-1/p62 in MM-F1 and MM-B1 cells, as compared to single treatments. The accumulation of p62 and LC3-II supports an inhibition of the autophagic flux (Fig. 6).

### Effects of GANT and AFA, alone or in combination, on the expression and activation of ErbB receptors (EGFR and ErbB2) and pro-survival signaling molecules (ERK, JNK, p38, Akt)

**Fig. 7:**
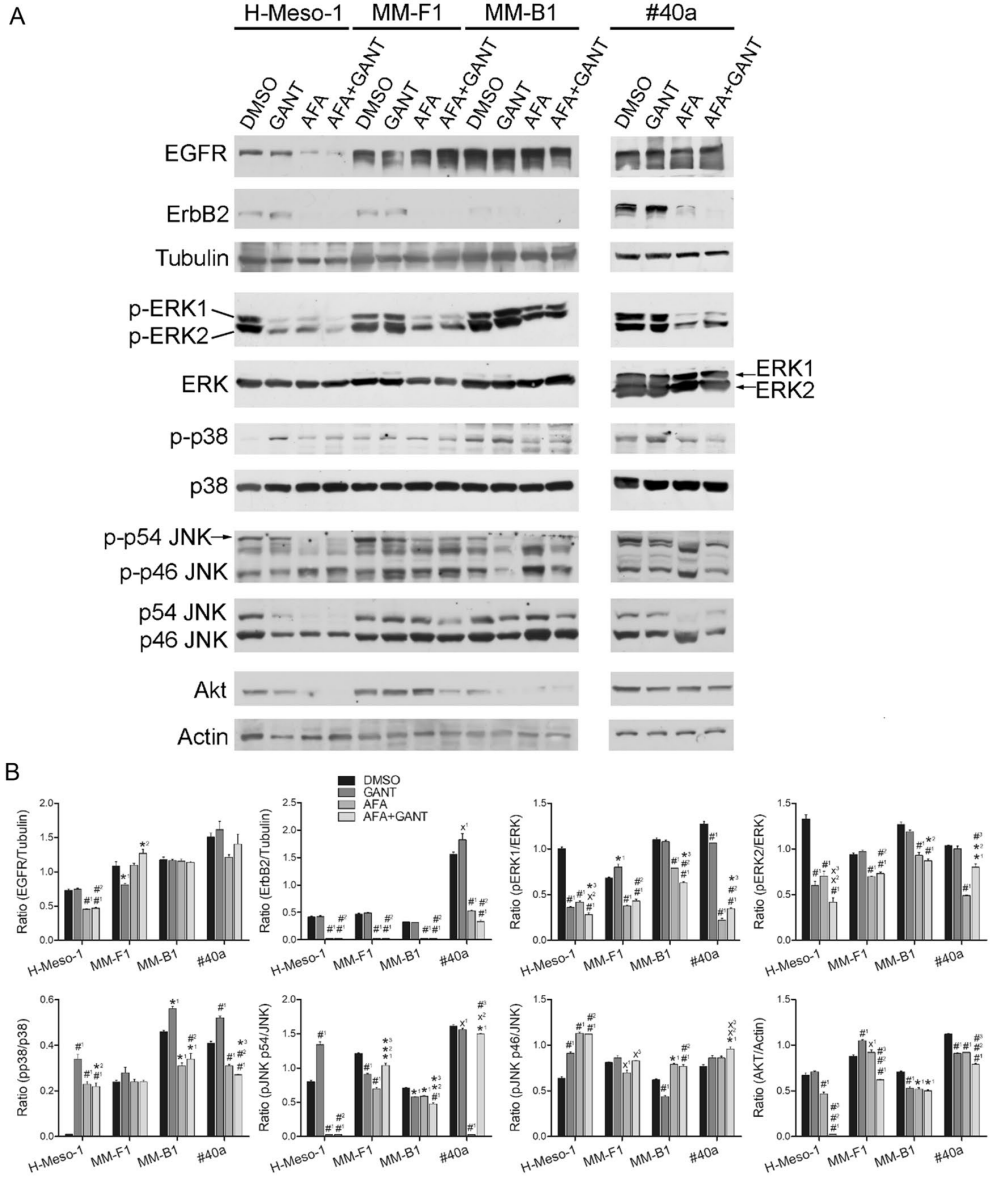
Effects of GANT and AFA, alone or in combination, on the expression and activation of signaling pathway molecules in MM cell lines. **A** Expression of EGFR, ErbB2 and Akt, expression of ERK1/2 and phospho-ERK1/2 (p-ERK1/2), p38 and phospho-p38 (p-p38), p54 and p46 JNK and phospho-p54 and -p46 JNK (p-p54 JNK, p-p46 JNK) was evaluated by Western blotting analysis following treatment of MM cells with GANT and AFA, alone or in combination, for 48 h. Tubulin and actin were used as internal controls. **B** Densitometric and statistical analysis are reported. The intensity of the bands was quantified using the ImageJ 1.53e software after blot scanning. The ratios of EGFR *vs* tubulin, ErbB2 *vs* tubulin, ERK1 and ERK2 *vs* their phosphorylated forms, p38 *vs* its phosphorylated form, p46 and p54 JNK *vs* their phosphorylated forms, Akt *vs* actin are shown. Statistical significance of the effects obtained with GANT and AFA, alone or in combination, was calculated *vs* those obtained in (1) DMSO-, (2) GANT- and (3) AFA-treated cells with one-way ANOVA (˟p ≤ 0.05, *p ≤ 0.01, ^#^p 0.001). Data are expressed as the mean ± SD of two independent experiments. Cropped images were used for Western Blotting

ErbB receptors are overexpressed in MM [16–18]. Thus, the expression of EGFR, ErbB2 and Akt, and the expression and activation of mitogen-activated protein (MAP) kinases such as ERK 1/2, p38 and c-Jun N-terminal Kinases (JNK) were analyzed by Western blotting (Fig. 7). AFA and AFA + GANT significantly reduced the expression of EGFR in H-Meso-1 cells and inhibited the expression of ErbB2 in all cell lines. No difference was found in EGFR and ErbB2 expression between AFA- and AFA + GANT-treated cells thus suggesting that AFA alone achieved the maximal effect in modulating the expression of these receptors. In addition, AFA and AFA + GANT treatments decreased ERK1 and ERK2 phosphorylation in all cell lines compared to DMSO-treated cells. GANT potentiated the reduction of ERK1 and ERK2 phosphorylation induced by AFA in the H-Meso-1 cell line (Fig. 7).

The phosphorylation of p38 was increased after GANT treatment in all cell lines with the exclusion of MM-F1, and by AFA and AFA + GANT treatments in H-Meso-1 cells. Conversely, AFA diminished p38 phosphorylation in MM-B1 and #40a cells. In these cells the effect of AFA was prominent, and the addition of GANT was not able to modify p38 phosphorylation levels. The levels of p38 phosphorylation remained unchanged upon different treatments in MM-F1 cell line (Fig. 7).

p54 JNK expression and phosphorylation were strongly inhibited by the treatment with AFA in H-Meso-1 and #40a cells compared to DMSO-treated cells. AFA inhibited p54 JNK phosphorylation in MM-F1 and MM-B1 cells as well. GANT treatment inhibited p54 JNK phosphorylation in MM-B1, MM-F1 and #40a cells. The addition of AFA to GANT did not further decrease p54 JNK phosphorylation. p46 JNK phosphorylation was increased by AFA in H-Meso-1 and MM-B1 cell lines. On the other hand, GANT inhibited p46 JNK phosphorylation in MM-B1 cell line, while AFA was more potent in sustaining p46 JNK phosphorylation in this cell line (Fig. 7).

AFA treatment diminished Akt expression in H-Meso-1 and MM-B1 cells. Similarly, GANT inhibited Akt expression in MM-B1 cells. However, both AFA and GANT when used alone increased Akt expression in MM-F1 cells, as compared to control. Surprisingly, when MM-F1 cells were treated simultaneously with AFA and GANT, Akt expression was significantly inhibited, as compared to treatment with AFA and GANT alone or DMSO. In addition, GANT potentiated the reduction of Akt expression induced by AFA in H-Meso-1 and #40a cells (Fig. 7).

### Effects of GANT and AFA, alone or in combination, on the Hh pathway

Among the signaling pathways, whose cross-talk plays an important role in neoplastic transformation, are those mediated by ErbB receptors and the Hh/GLI signaling cascade [32]. First, to monitor the effects of GANT, AFA and AFA + GANT treatments after 24 h on MM cells, basal levels of the Hh pathway activity were evaluated by evaluating  GLI1, GLI2, and Ptch1 mRNA levels in untreated MM cells. Indeed, evaluation of GLI1, GLI2 and Ptch1 mRNA levels is a sensitive readout for monitoring GANT inhibitory activity on the Hh/GLI pathway [56].

**Fig. 8:**
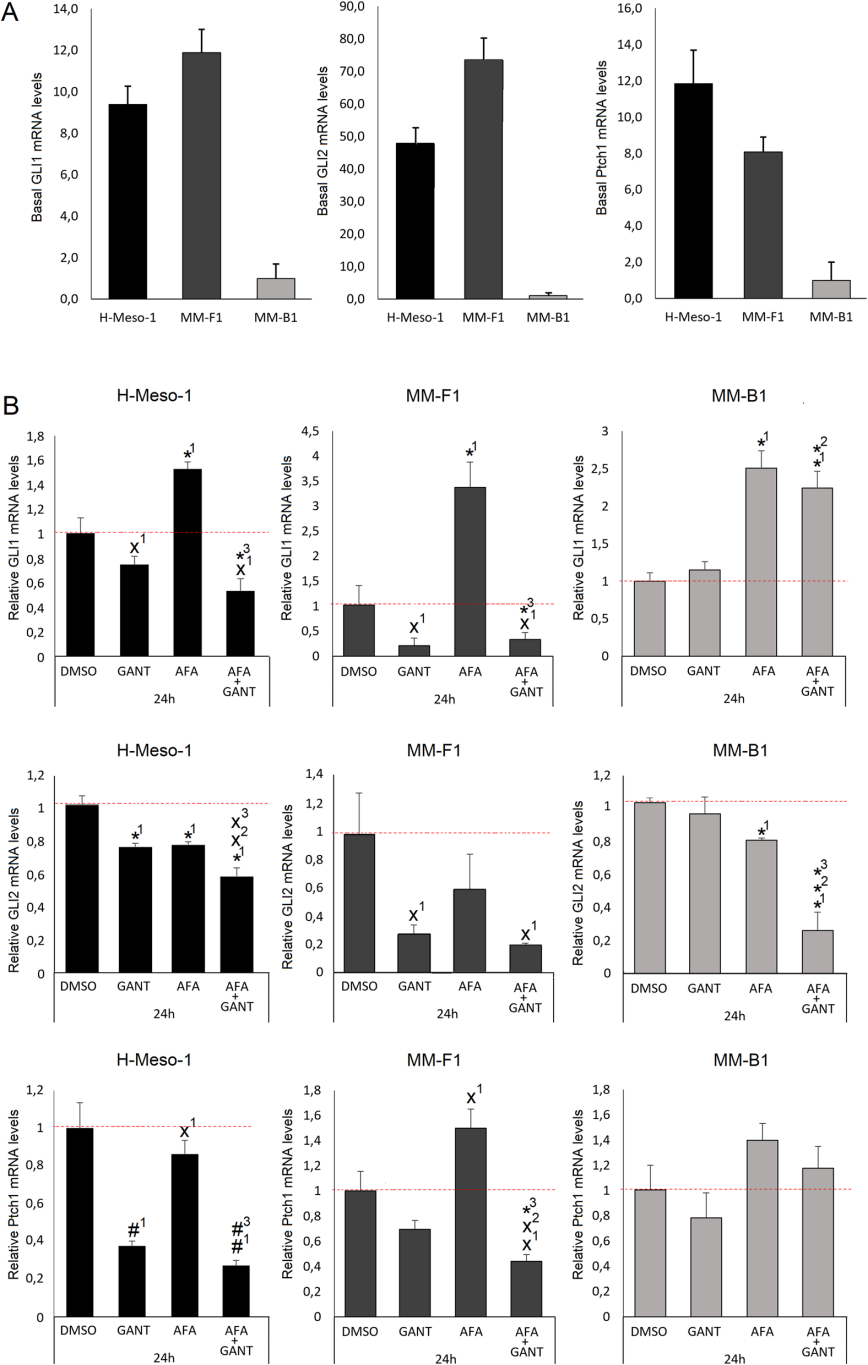
Effects of GANT and AFA, alone or in combination, on the Hh pathway. **A** Real-time q-PCR was performed to assess basal levels of Hh pathway activity in MM cell lines. **B** GLI1, GLI2, and Ptch1 mRNA levels in MM cell lines treated with AFA 10 μM, GANT 20 μM, AFA 10 μM + GANT 20 μM, or DMSO for 24 h. GLI1, GLI2, and Ptch1 mRNA expression levels are shown after normalization to the controls. The results are expressed as the mean ± SD of three independent experiments. Statistical significance of the effects obtained with GANT and AFA, alone or in combination, was calculated *vs* those obtained in (1) DMSO-, (2) GANT-, and (3) AFA-treated cells (˟p ≤ 0.05, *p ≤ 0.005, ^#^p ≤ 0.0005) with a two-tailed Student’s t test

As shown in Fig. 8A, while H-Meso-1 and MM-F1 cells exhibited a significative Hh pathway basal activity, MM-B1 cells showed very low basal levels of GLI1, GLI2, and Ptch1, suggesting that the pathway may be active at very low levels. In agreement with this hypothesis, GANT significantly reduced GLI1 and GLI2 mRNA levels in H-Meso-1 and MM-F1 cell lines while it had no effects on MM-B1 cells as compared to DMSO-treated cells (Fig. 8B). Interestingly, treatment with AFA increased the levels of GLI1 mRNA in all cell lines as compared to DMSO-treated cells, while it reduced the levels of GLI2. Again, the addition of GANT in the combined treatment inhibited AFA-induced GLI1 mRNA overexpression in H-Meso-1 and MM-F1 cells, but not in MM-B1 cells, as compared to control cells (Fig. 8B). Remarkably, the combined AFA + GANT treatment improved GLI2 inhibition not only in H-Meso-1 and MM-F1 cell lines, but also in the low-GLI2 expressing MM-B1 cells. The effects of the different treatments on the Hh pathway were further confirmed by analyzing the modulation of the expression levels of Ptch1 upon treatments in H-Meso-1 and MM-F1 cells (Fig. 8B). While AFA increased the Hh pathway activity, as compared to control cells, the combined treatment of AFA + GANT induced a reduction of the pathway activity below the basal level (Fig. 8B). The different basal Hh pathway activity in the different cell lines could explain differential effects of GANT and AFA + GANT in the three cell lines.

### In vivo effect of treatment with GANT and AFA, used alone or in combination, on the growth of MM #40a cells in syngeneic C57BL/6 mice

To evaluate the in vivo antitumor effect of GANT and AFA, alone or in combination, on the growth of MM cells in syngeneic C57BL/6 mice, groups of mice (n = 8/group) were i.p. inoculated with 1 × 10^6^ syngeneic MM cells (#40a) and i.p. treated with AFA (25 mg/kg), GANT (50 mg/kg), or AFA (25 mg/kg) + GANT (50 mg/kg) dissolved in PBS + DMSO, or with the vehicle alone (PBS + DMSO). Treatments began simultaneously with the inoculation of cells and were repeated twice a week. Since i.p. inoculation of #40a cells induces ascites, in order to monitor the growth of cancer cells, mice abdominal circumference was measured before the inoculation of the cells and then every week. Mice were sacrificed at the first sign of stress.

**Fig. 9:**
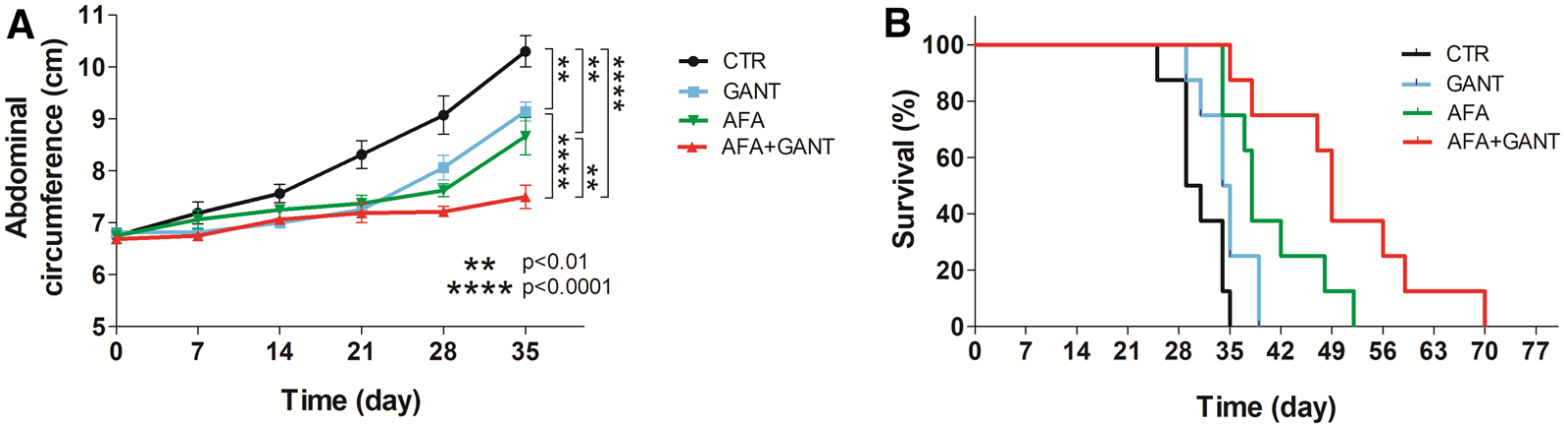
GANT and AFA, used alone or in combination, reduced tumor growth and increased survival in C57BL/6 mice i.p. transplanted with MM #40a cells. **A** Differences in mean abdominal circumferences among mice treated with GANT, AFA, AFA + GANT or PBS + DMSO (CTR). **B** Differences in the mean survival duration of mice treated with GANT, AFA, AFA + GANT or PBS + DMSO (CTR). The numbers of treated mice are reported in the “Materials and Methods”

After 21 days of treatment, mice treated with GANT, AFA, and AFA + GANT showed a significantly lower increase in the abdominal circumference, as compared to control mice (CTR) treated with the vehicle alone (GANT 7.3 *vs* CTR 8.3 cm, p = 0.002; AFA 7.4 *vs* CTR 8.3 cm, p = 0.009; AFA + GANT 7.2 *vs* CTR 8.3 cm, p = 0.004) (Fig. 9A). However, no differences were shown between single and combined treatments. The lower increase of the abdominal circumference of mice treated with GANT, AFA and AFA + GANT compared to control mice was also maintained at 28 days (GANT 8.1 *vs* CTR 9.1 cm, p = 0.035; AFA 7.6 *vs* CTR 9.1 cm, p = 0.002; AFA + GANT 7.2 *vs* CTR 9.1 cm, p = 0.0004) and at 35 days (GANT 9.1 *vs* CTR 10.3 cm, p = 0.006; AFA 8.7 *vs* CTR 10.3 cm, p = 0.008; AFA + GANT 7.4 *vs* CTR 10.3 cm, p = 0.00003) (Fig. 9A). Of note, at 28 and 35 days, mice treated with AFA + GANT showed a significantly lower abdominal circumference than mice treated with GANT (p = 0.009 at 28 days; p = 0.0001 at 35 days) or AFA (p = 0.03 at 28 days; p = 0.02 at 35 days) (Fig. 9A).

AFA + GANT treatment induced a significant increase in the mean survival of mice as compared to control mice (49 *vs* 30 days, p = 0.05). The risk of tumor growth in CTR-treated mice was 21.00 times greater than that of AFA + GANT-treated mice (Fig. 9B and Table 1). The increase in the mean survival of AFA + GANT-treated mice was also significant compared to GANT- (49 *vs* 34.5 days, p = 0.002) and AFA-treated mice (49 *vs* 38 days, p = 0.05). The risk of tumor growth in GANT-treated or AFA-treated mice was found to be 9.369 and 3.746 times greater than that of AFA + GANT-treated mice respectively (Fig. 9B and Table 1).

**Table 1:** Analysis of the survival of C57BL/6 mice after treatment with GANT, AFA, and AFA + GANT by the log-rank test (Mantel-Cox)

			Hazard Ratio(HR)	95% HR Confidence intervals	Median Survival (day)	p-value
CTR	*vs*	GANT	3.761	(1.016, 13.91)	30 *vs* 34.5	0.05
CTR	*vs*	AFA	12.58	(2.908, 54.45)	30 *vs* 38	0.002
CTR	*vs*	AFA + GANT	21.00	(4.548, 96.91)	30 *vs* 49	0.0003
GANT	*vs*	AFA	3.092	(0.9098, 10.51)	34.5 *vs* 38	0.04
GANT	*vs*	AFA + GANT	9.369	(2.335, 37.58)	34.5 *vs* 49	0.002
AFA	*vs*	AFA + GANT	3.746	(1.106, 12.69)	38 *vs* 49	0.05

CTR: control (PBS + DMSO); GANT: GANT-61; AFA: Afatinib; AFA + GANT: Afatinib + GANT-61

Our results indicate that AFA + GANT treatment is superior to single treatments in impairing the growth of i.p. transplanted MM #40a cells.

## Discussion

MM is a rare orphan aggressive neoplasia with low survival rates [1, 2]. ErbB receptors and Hh signaling pathways are deregulated in MM [16–18, 57]. Thus, molecules involved in these signaling pathways could be used to develop targeted therapy approaches.

AFA is a second-generation TKI, which irreversibly binds and inhibits the tyrosine kinase activity of members of the ErbB family including EGFR, ErbB2/HER2 and ErbB4. AFA targets both mutated and wild-type EGFR [58]. AFA has been employed in several clinical trials in the last years for the treatment of NSCLC, head and neck squamous cell carcinoma, breast cancer, colorectal cancer, brain cancer, prostate cancer, gastric cancer. Upon TKIs treatment acquired resistance inevitably occurs in cancer patients and remains a biological challenge [59–64].

Mechanisms of resistance to EGFR inhibition by TKIs could be due to a simultaneous activation of alternative signaling pathways [22, 23].

GANT is a small molecule with inhibitory activity on the transcription factors GLI1 and GLI2, showing a high specificity for the Hh-mediated transduction pathway. Although several inhibitors of the Hh pathway targeting SMO are presently available (e.g. Vismodegib, Sonidegib), we chose to employ the GLI inhibitor GANT, since, as compared to SMO, GLI proteins have been indicated as more potent therapeutic targets in different tumors, including MM [65, 66]. Indeed, it is known that GLI can be activated also via SMO-independent non-canonical mechanisms involving cross-talks between different oncogenic signaling pathways, among which the ErbB pathway, leading to resistance to SMO inhibitors [27, 32, 66]. GANT has been shown to exert an anti-cancer activity in vitro and in vivo on different types of cancer, by inhibiting the expression of both GLI and Ptch [27, 67–70].

The effect of AFA was previously analyzed in MM cells alone or in combination with other drugs. Specifically, the effect of AFA was evaluated in association with crizotinib, a drug used in NSCLC and capable of inhibiting MET, Alk and ROS-1 kinases. Another study on MM cells evaluated the synergistic use of AFA and trastuzumab (anti-HER2 monoclonal antibody). An increase in the antibody-dependent cellular cytotoxicity (ADCC) mechanism has been observed, with promising anticancer effects [71]. In addition, GANT is reported to induce apoptosis following oxidative stress in MM cells in vitro [72].

Accumulating evidence indicates that a complex interplay can occur between ErbB receptors and Hh signaling. Moreover, cooperation of EGFR signaling with Hh/GLI was demonstrated to promote cancer cells transformation and proliferation [32]. To date, there are no studies reporting the combined use of AFA and GANT for cancer treatment.

Accordingly, the aim of this study was to evaluate the in vitro effects of AFA and GANT, used alone or in combination, on growth, cell cycle regulation, activation of cell death and autophagy, modulation of molecules involved in signaling pathways in three human MM cell lines, having different histotype (MM-B1, biphasic phenotype; MM-F1, fibromatous phenotype; H-Meso-1, epithelioid phenotype), and in a murine epithelioid MM cell line (#40a). To our knowledge, this is the first report analyzing the effect of the combination of AFA and GANT on MM cells.

Our results show that treatment with AFA and GANT, used alone and in combination, significantly inhibited the growth of MM cells in a dose- and time-dependent manner. The growth inhibition induced by the combined treatment AFA + GANT was superior to the effect obtained employing single treatments.

When analyzing the effect of single treatments on cells growth and death and on the induction of apoptosis, it was observed that the cell lines most sensitive to the drugs were H-Meso-1 and MM-B1, with epithelioid and biphasic histotypes, respectively. The fibromatous cell line (MM-F1) was less sensitive to single treatments when analyzing the same parameters. The behavior of the mouse epithelioid cell line #40a treated with single drugs was similar to that of the H-Meso-1 cell line. As regards the effect of the two single treatments on cell proliferation, it must be considered that AFA was certainly more powerful than GANT in its anticancer effects on MM. However, it must be highlighted that when the two drugs were used at the highest concentrations the combination with GANT further increased the AFA-induced inhibition on cell proliferation. Based on the analysis of drug interaction, the combination of AFA and GANT had synergistic effects in reducing MM cell survival depending on both the dose and cell line tested. The synergistic effect was more evident in MM-B1 and MM-F1 cell lines when the two drugs were used at high concentrations. When analyzing the effects of the combined treatment on the human H-Meso-1 and mouse #40a cell lines, the overall effect was instead less than additive, but still superior to that obtained with AFA alone. Our findings support the use of the Hh/GLI pathway inhibitor in combination with the ErbB receptors pathway inhibitor for the treatment of MM. Worthy of note, based on the results obtained on the MM-F1 cell line, which was the less sensitive to treatment with AFA alone, the combined treatment may be beneficial to those patients that are poorly responsive to the single treatment with an ErbB receptors pathway inhibitor.

To corroborate the in vitro findings, C57BL/6 mice were i.p. inoculated with syngeneic MM cells (#40a) and i.p. treated with AFA, GANT, or AFA + GANT. Our results show that AFA, GANT, and AFA + GANT treatments were able to significantly interfere with the in vivo tumor growth of mouse MM cells transplanted into the peritoneum compared to control mice. Moreover, AFA, GANT, and AFA + GANT were able to induce a significant increase in the mean survival and a reduction of the tumor volume compared to control mice. Remarkably AFA + GANT were more effective in increasing the mean survival and in reducing the abdominal circumference of mice compared to GANT and AFA alone.

In order to understand whether and how GANT treatment could interfere with the signaling pathways targeted by AFA, we analyzed their effects alone or in combination on the activation of autophagy and signaling pathways mediated by ErbB receptors and Hh. From these studies it emerges that some of the molecular events induced by the drug treatments were similar across the different cell lines, whereas other effects were more variable and cell-line dependent.

For instance, on the whole AFA and GANT appeared to inhibit autophagy on all the cell lines tested, when used alone or in combination. Indeed, the single and combined treatments induced a concurrent increase of the autophagosome marker LC3-II and the selective autophagy substrate p62 in H-Meso-1, MM-B1 and #40a cell lines, consistent with an inhibition of the autophagic flux. Similar effects were obtained with GANT and AFA + GANT also in MM-F1 cells, where however the AFA single treatment had no effect on LC3-II levels. On the other hand, AFA was still able to increase the levels of p62 in MM-F1 cells, indicating that an inhibition of autophagy may be induced by AFA also in this cell line. According to these findings, the increased antitumor effect exerted by the combined treatments might be due to an impaired ability of the cells to cope with cellular stress via autophagy. By the way, the functional significance of Beclin-1 fluctuations induced by the single drugs appears controversial, since AFA and GANT had variable, cell line-dependent effects on Beclin-1 levels when used alone. On the other hand, the combined treatment decreased Beclin-1 in every cell line except MM-F1.

Next, the effect of AFA on signaling mediated by ErbB receptors was analyzed. AFA downregulated ErbB2 levels in all cell lines and EGFR levels in H-Meso-1. Consistent with the effect exerted on ErbB receptors, AFA inhibited the activation of the downstream effectors ERK1 and ERK2, which primarily transduce proliferative signals [32], and p54 JNK in all human and mouse cell lines. Of note, GANT potentiated the inhibitory effect of AFA on ERK1 and ERK2 phosphorylation in H-Meso-1 cells and on ERK1 in MM-B1 cells. On the other hand, as regards p38, the effect of AFA and GANT were opposite or similar, depending on the cell line. AFA decreased p38 phosphorylation in MM-B1 and #40a cells, whereas GANT had an opposite effect. However, the effect of AFA was prominent, and the addition of GANT was not able to modify the level of p38 phosphorylation. Conversely, both AFA and GANT promoted p38 phosphorylation in H-Meso-1 cells. The p38 MAPK plays a dual role as a promoter of cell death or survival depending on the type of stimulus and cell [73–75]. Similarly, it has been reported that JNK has a dual role in regulating both apoptosis and survival of cancer cells [74–76]. Several studies have demonstrated that p46 JNK and p54 JNK exhibit opposite functions in the regulation of cell survival and tumor development. In particular, some studies have reported that p46 JNK triggers a death signal, whereas p54 JNK induces cell survival. However, other studies have reported an opposite effect. Thus, the main regulator of cell survival between p46 and p54 JNK remains to be determined [76]. In addition, a crosstalk between p38 and JNK in regulating autophagy and apoptosis induced by DNA damage has been reported [77]. In our study, AFA alone potently inhibited the activity of p54 JNK in all cell lines. However, the addition of GANT to AFA appeared to affect p54 JNK phosphorylation in a variable, and once more cell line-dependent manner.

As far as the effect of the treatments on Akt are concerned, a more consistent trend was instead observed across the different cell lines. In fact, AFA inhibited Akt expression in all cell lines tested with the exception of MM-F1. However, the combined AFA + GANT treatment significantly reduced the expression of Akt in the MM-F1 cell line where AFA alone did not affect this kinase expression. In addition, Akt expression was significantly decreased with the combined treatment *vs * AFA alone in H-Meso-1 and in the mouse #40a cell line. Therefore, as compared with the decrease of Akt obtained with the single agents, the combination of AFA + GANT was able to further reduce the expression of this pro-survival kinase in three out of four MM cell lines.

The decrease of Akt expression by AFA plus GANT appeared to inhibit pro-survival signals and induce apoptosis in MM cells. Accordingly, we found that AFA increased the Bax/Bcl-2 ratio in H-Meso-1, MM-B1, #40a cell lines but not in MM-F1 cell line. It is worth noting that AFA was able to increase apoptosis in the MM-F1 cell line and this effect was potentiated by GANT. The activation of apoptosis in AFA and AFA + GANT-treated MM-F1 cells was corroborated by the increase in γH2AX.

It is well known that PI3K/Akt can be regulated by EGFR/ErbB2 receptors and by Hh/GLI signaling pathways [32]. In turn, PI3K/Akt regulate the activation of GLI1 in melanoma and other cancer cells. Thus, Akt is essential for GLI-dependent activation of Hh signaling [78–80]. In addition, EGFR/ErbB2 signaling synergizes with GLI1/2 to selectively induce the transcription of target genes through stimulation of RAS/RAF/MEK/ERK signaling [81].

Concerning Hh signaling, we observed that GANT reduced GLI1, GLI2 and Ptch1 mRNA levels in H-Meso-1 and MM-F1 cells. However, we could not detect a similar inhibitory effect in MM-B1 cells, probably due to the low basal Hh pathway activity in this cell line. AFA decreased GLI2 mRNA levels, as well, but it increased GLI1 mRNA levels in all the cell lines analyzed, and Ptch1 mRNA levels in MM-F1 cells. This finding suggests that the inhibition of the EGFR pathway can induce the activation of alternative pathways in MM cells such as the Hh/GLI1 pathway, that could be activated most likely through a non-canonical pathway.

In turn, it has been demonstrated that activation of the Hh pathway can promote resistance to EGFR inhibitors, possibly via the induction of tumor cell epithelial-to-mesenchymal transition [82]. Accordingly, in the Hh-dependent cell lines H-Meso-1 and MM-F1, AFA treatment appears to trigger an “escape” mechanism through the overactivation of Hh/GLI1. In this respect, the combination with a GLI inhibitor may be beneficial, since it may allow to compensate for the stimulatory effect of AFA on Hh/GLI1 signaling. Indeed, the addition of GANT to AFA was able to counteract the AFA-mediated induction of GLI1 and Ptch1 expression. Of note, the reduction of GLI1 mRNA levels can explain the additive inhibitory effect exerted by the combination of GANT and AFA on Akt expression, since both EGFR/ErbB2 receptors and Hh/GLI signaling pathways can mediate the inhibition of this kinase.

Current multimodal therapies including surgery, chemotherapy and radiotherapy, have improved the quality of life of MM patients, but the prognosis for this tumor still remains poor.

Preclinical studies and clinical trials have addressed the therapeutic efficacy of many new antitumor agents on MM. Intracavitary drug administration has also been investigated as a mean of improving the effectiveness of different agents via local delivery at the tumor site [15].

Among the angiogenesis-targeting agents, the anti-VEGF monoclonal antibody Bevacizumab has been shown to improve overall survival in MM patients when combined with standard chemotherapy [15]. Conversely, other anti-angiogenic agents, including Axitinib (an anti VEGFR TKI), Sorafenib (a TKI targeting VEGFR2/3, platelet-derived growth factor receptor (PDGFR) and rapidly accelerated fibrosarcoma (RAF)/c-KIT), and Imatinib mesylate (a TKI targeting BCR-ABL, c-KIT, and PDGFR) failed to show clinical efficacy on MM [83–87].

The MEK and p110β/PI3K inhibitors Seleumetinib and AZD8186, currently being tested in clinical trials on different cancer types, have been reported to increase survival and display a low toxicity profile in a murine model of sarcomatoid MM [88]. A strong EGFR activation associated with HER2, HER3, Axl, and MET co-activation, mediated mainly by receptor heterodimerization and autocrine-paracrine loops induced by the expression of their cognate ligands, has been demonstrated in patients with peritoneal MM. These results support the possible use of different targeted therapy approaches to inhibit the pathways activated by these receptors [89].

The combination of different targeted therapy and immunotherapy approach, using immune checkpoint blockade regimens can potentially deliver new opportunities to improve anti-cancer treatments for MM patients [90].

In the present study we provide evidence that the combined use of two drugs capable of counteracting the activation of two different, aberrantly activated signaling pathways (ErbB receptors and Hh) could be a useful tool to reduce the growth of MM cells.

## Conclusions

Our study demonstrates that combined treatment with two inhibitors counteracting the activity of two different signaling pathways involved in neoplastic transformation and progression, such as those activated by ErbB and Hh, is more effective than the single treatments in reducing MM growth in vitro and in vivo. This study may have clinical implications for the development of targeted therapy approaches for MM.

## Data Availability

Not applicable.
